# Risk factors for pancreas and lung neuroendocrine neoplasms: a case–control study

**DOI:** 10.1007/s12020-020-02464-5

**Published:** 2020-08-31

**Authors:** Luca Giraldi, Alessia Vecchioni, Greta Carioli, Mirna Bilotta, Stefano La Rosa, Andrea Imperatori, Marco Volante, Maria Pia Brizzi, Frediano Inzani, Gianluigi Petrone, Giovanni Schinzari, Antonio Bianchi, Stefano Margaritora, Sergio Alfieri, Carlo La Vecchia, Stefania Boccia, Guido Rindi

**Affiliations:** 1 Section of Hygiene, Department of Life Sciences and Public Health, Università Cattolica del Sacro Cuore, Roma, Italia; 2 Department of Clinical Sciences and Community Health, University of Milan, Milan, Italy; 3 Section of Anatomic Pathology, Department of Life Sciences and Public Health, Università Cattolica del Sacro Cuore, Roma, Italia; 4 Department of Medicine and Surgery, University of Insubria, Varese, Italy; 5 Department of Oncology, University of Turin at San Luigi Gonzaga Hospital, Orbassano, Italy; 6 Department of Oncology, Fondazione Policlinico Universitario A. Gemelli IRCCS, Roma, Italia; 7 Department of Endocrinology, Fondazione Policlinico Universitario A. Gemelli IRCCS, Roma, Italia; 8 Department of Thoracic Surgery, Fondazione Policlinico Universitario A. Gemelli IRCCS, Roma, Italia; 9 Digestive Surgery Unit, Fondazione Policlinico Universitario A. Gemelli IRCCS, Roma, Italia; 10 Section of Hygiene, Department of Woman and Child Health and Public Health, Fondazione Policlinico Universitario A. Gemelli IRCCS, Roma, Italia; 11 Section of Anatomic Pathology, Department of Woman and Child Health and Public Health, Fondazione Policlinico Universitario A. Gemelli IRCCS, Roma, Italia; 12 Roma European NeuroEndocrine Tumor Society (ENETS) Center of Excellence, Roma, Italia; 13 Present Address: Institute of Pathology, Lausanne University Hospital and University of Lausanne, Lausanne, Switzerland

**Keywords:** Lung, Pancreas, Neuroendocrine neoplasia, Case–control, Risk factor, OR

## Abstract

**Purpose:**

Neuroendocrine neoplasia (NEN) has been displaying an incremental trend along the last two decades. This phenomenon is poorly understood, and little information is available on risk factor for neuroendocrine neoplasia development. Aim of this work is to elucidate the role of potentially modifiable risk factors for pancreatic and pulmonary NEN.

**Methods:**

We conducted a case–control study on 184 patients with NEN (100 pancreas and 84 lung) and 248 controls. The structured questionnaire included 84 queries on socio-demographic, behavioral, dietary and clinical information.

**Results:**

Increased risk was associated with history of cancer (“other tumor”, lung OR = 7.18; 95% CI: 2.55–20.20 and pancreas OR = 5.88; 95% CI: 2.43–14.22; “family history of tumor”, lung OR = 2.66; 95% CI: 1.53–4.64 and pancreas OR = 1.94; 95% CI: 1.19–3.17; “family history of lung tumor”, lung OR = 2.56; 95% CI: 1.05–6.24 and pancreas OR = 2.60; 95% CI: 1.13–5.95). Type 2 diabetes mellitus associated with an increased risk of pancreatic NEN (OR = 3.01; 95% CI: 1.15–7.89).

**Conclusions:**

Besides site-specific risk factors, there is a significant link between neuroendocrine neoplasia and cancer in general, pointing to a shared cancer predisposition.

## Introduction

Neuroendocrine neoplasms (NENs) are a heterogeneous group of neoplasms in different anatomical sites [1]. Most of NENs occur in the small intestine and pancreas, followed by lung and the respiratory system and by colon and rectum [2, 3]. The incidence of these tumors is usually lower than 5/100,000 [4–7]. NENs typically have a long survival that results in a prevalence rate of 48/100,000 [2]. The epidemiology of NENs is changing, since in recent years there was an increased incidence only partially explained by better diagnostic techniques [8]. Increased trend have been shown in various populations and in different anatomical sites for both low-grade and high-grade NENs [9–13].

Several risk factors have been associated with the risk of developing NENs. A systematic review and meta-analysis published in 2016 indicates family history of cancer as the main risk factor for NENs of the pancreas, rectum, stomach and lung [14]. Other important risk factors referring to individual’s behavior such as cigarette smoking and alcohol drinking, mainly affected lung and pancreas (ibidem).

We conducted a case–control study in three Italian centers with the aim to elucidate the role of potentially modifiable risk factors for the most prevalent and aggressive NENs, pancreatic and pulmonary.

## Materials and methods

### Participants and study design

The design was prospective case–control study. Study cases were patients with NENs of the pancreas and of the lung. Cases were enrolled in three different Italian hospitals: Policlinico Agostino Gemelli (Rome), Ospedale San Luigi Gonzaga (Orbassano) and Ospedale di Circolo (Varese). Patients with pancreatic or lung NENs were included. Details on tumor functionality were not collected. Cases with multiple endocrine neoplasia (MEN) were excluded. Controls were randomly recruited among healthy individuals admitted at the same hospital of the cases, in the same period, for non-neoplastic conditions. Individuals with severe neurological or psychiatric disorders were excluded. Enrollment lasted from 2014 to 2017.

Cases and controls were interviewed by trained interviewers using a questionnaire including information on socio-demographic, behavioral, dietary, and clinical information. The variables were: demographic features; tumor history; smoke habit; alcohol consumption; diet; exercise and lifestyle; medical history and gynecological/obstetric data (for women), investigated with 83 specific queries in Italian language. All queries to patients referred to the time prior to the diagnosis of neuroendocrine neoplasia. The questionnaire was validated by the local ethics committee and adopted in each center. All the information was collected at each participating center and shared with the coordinating center at the Fondazione Policlinico Universitario IRCCS-Università Cattolica del Sacro Cuore where data were checked and corrected for inconsistencies. Participation rate was calculated as the proportion of respondents who completed the interview.

### Variables definition

Tumor grade was defined according to the WHO classification of 2017 and 2019 for pancreatic NEN [15, 16] and WHO 2015 for lung NEN tumors [17]. Smoking and alcohol including information on status (never, former, current) and to the intensity (number of pack-years and number of drinks per day) respectively. The definition of smoking/alcohol drinking/status was as follows: participants who were smoking (or drinking) until one year before the diagnosis for cases and interview for controls were defined as current smokers (or drinkers); participants who quitted smoking (or drinking) more than one year before the diagnosis for cases and the interview for controls were defined as former smokers (or drinkers). Diet was defined using the current Italian dietary definition and the Mediterranean diet score [18–20]. Exercise and life-style items were defined as published [21]. Participants body mass index (BMI) was categorized according to the World Health Organization cutoff points [22].

### Statistical analysis

Descriptive statistics were used to describe the participant’s characteristics. Comparison between the distribution of selected variables between cases and controls was conducted using Student *t* test or Mann–Whitney test for continuous variables and Chi-squared of Fisher exact test for categorical variables, where appropriate. Adjusted Odds Ratios (ORs) and the 95% Confidence Intervals (CI) for the association between selected variables and the risk of lung NEN and pancreatic NEN were estimated using a multivariable logistic regression model. The following terms were included in the model as confounders: age, gender, family history of cancer and smoking intensity. In order to minimize the potential confounder due to the inclusion of prevalent cases [23], a separate analysis was conducted including only the incident cases, defined as the cases that were interviewed within one year of being diagnosed with NENs. Two sensitivity analysis were conducted as follows: (1) an analysis was conducted including age-matched cases and controls and (2) an analysis was conducted that was limited to TC and AC cases. All tests were two-sided and a *p* value less than 0.05 was considered statistically significant. Statistical analysis was conducted using Stata software (StataCorp. 2013. Stata Statistical Software: version 13. College Station, TX: StataCorp LP).

### Ethic Committee approval

Subjects gave written informed consent to this study. The study protocol was approved by the Ethic Committee of Fondazione Policlinico Universitario A. Gemelli – Università Cattolica del Sacro Cuore (N. 27229/13).

## Results

Participation rate was 98% for all study participants. A total of 184 cases and 248 controls were included in the study, the majority of which were recruited in the center of Rome (81.0% of the cases and 96.4% of the controls).

Table 1 shows the distribution of cases with pancreatic NEN, lung NEN cancer and controls according to selected variables. A total of 100 cases (54.3%) had pancreatic NEN. The age distribution of controls was significantly younger compared to both lung (*p* < 0.001) and pancreatic NEN (*p* < 0.001).

**Table 1:** Distribution of selected covariates among cases and controls

Characteristics	Controls		Cases						
	*n*	%	Lung NEN			Pancreatic NEN			
			*n*	%	*p* value^a^	*n*	%	*p* value^b^	*p* value^c^
Total	248	100.0	84	45.7		100	54.3		
Case type									0.580
Incident	–	–	57	67.9	–	45	45.0	–	
Prevalent	–	–	14	16.7	–	14	14.0	–	
Missing	–	–	13	15.5	–	41	41.0	–	
Age (years)					**<0.001**			**<0.001**	0.274
≤45	106	42.7	18	21.4		18	18.0		
46–60	67	27.0	21	25.0		36	36.0		
>60	75	30.2	45	53.6		46	46.0		
Missing	0	–	0	–		0	–		
Gender					**0.034**			0.331	0.306
Male	100	40.3	45	53.6		46	46.0		
Female	148	59.7	39	46.4		54	54.0		
Missing	0	–	0	–		0	–		
Height^d^	167.3^**d**^	10.8^**d**^	169.2^**d**^	7.5^**d**^	0.121	168^**d**^	7.9^**d**^	0.774	0.157
Weight^d^	70.4^**d**^	14.8^**d**^	75.0^**d**^	14.5^**d**^	**0.013**	75.8^**d**^	14.7^**d**^	**0.002**	0.738
BMI					0.327			**0.033**	0.589
Underweight	12	4.8	3	3.6		3	3.0		
Normal weight	69	27.8	21	25.0		17	17.0		
Overweight	135	54.4	43	51.2		58	58.0		
Obese	30	12.1	17	20.2		22	22.0		
Missing	2	0.8	0	–		0	–		
Education level					0.089			0.159	0.702
Low	80	32.3	38	45.2		42	42.0		
Medium	98	39.5	31	36.9		35	35.0		
High	58	23.4	14	16.7		22	22.0		
Missing	12	4.8	1	1.2		1	1.0		
Smoking status					**0.028**			0.120	**0.002**
Never	122	49.2	28	33.3		59	59.0		
Former	75	30.2	37	44.0		29	29.0		
Current	51	20.6	19	22.6		12	12.0		
Missing	0	–	0	–		0	–		
Smoking intensity					**<0.001**			0.255	**<0.001**
Never	122	49.2	28	33.3		59	59.0		
≤15 pack-years	66	26.6	17	20.2		23	23.0		
>15 pack-years	56	22.6	38	45.2		17	17.0		
Missing	4	1.6	1	1.2		1	1.0		
Alcohol status					0.464			0.301	0.460
Never	127	51.2	37	44.0		51	51.0		
Former	10	4.0	5	6.0		8	8.0		
Current	111	44.8	42	50.0		41	41.0		
Missing	0	–	0	–		0	–		
Alcohol intensity					0.259			0.631	0.633
Never	127	51.2	37	44.0		51	51.0		
≤1 drink per day	81	32.7	27	32.1		29	29.0		
>1 drink per day	40	16.1	20	23.8		20	20.0		
Missing	0	–	0	–		0	–		
Diabetes 1					0.852			0.133	0.348
No	240	96.8	82	97.6		94	94.0		
Yes	5	2.0	2	2.4		5	5.0		
Missing	3	1.2	0	–		1	1.0		
Diabetes 2					0.054			**0.002**	0.416
No	238	96.0	77	91.7		88	88.0		
Yes	8	3.2	7	8.3		12	12.0		
Missing	2	0.8	0	–		0	–		
Other tumor					**<0.001**			**<0.001**	0.845
No	238	96.0	68	81.0		79	79.0		
Yes	8	3.2	16	19.0		20	20.0		
Missing	2	0.8	0	–		1	1.0		
Family history of tumor					**<0.001**			**0.001**	0.270
No	146	58.9	27	32.1		40	40.0		
Yes	102	41.1	57	67.9		60	60.0		
Missing	0	–	0	–		0	–		
Family history of lung cancer				**<0.01**			**<0.01**	0.956	
No	236	95.2	72	85.7		86	86.0		
Yes	12	4.8	12	14.3		14	14.0		
Missing	0	–	0	–		0	–		

Text in bold means significant *p* value

*–* not computable, *NEN* neuroendocrine neoplasm

^a^*p* value from chi-square or *t*-test for comparison between lung cases and controls

^b^*p* value from chi-square or *t*-test for comparison between pancreas cases and controls

^c^*p* value from chi-square or *t*-test for comparison between pancreas cases and lung cases

^d^Data are mean, standard deviation

**Fig. 1:**
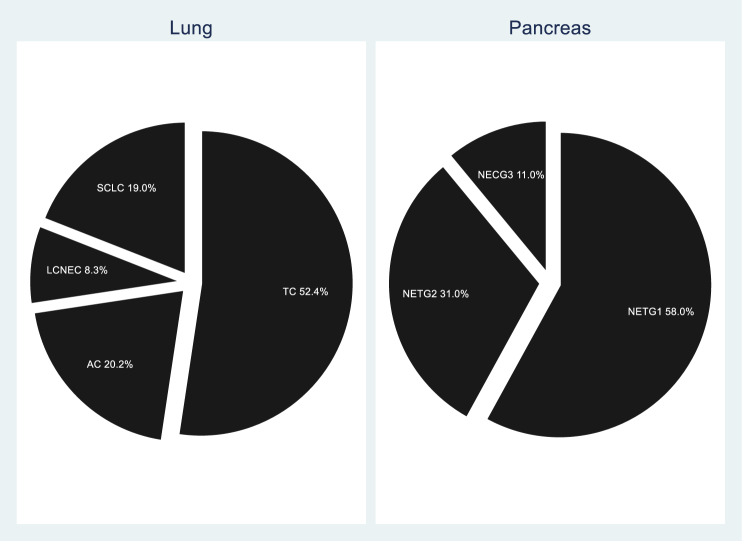
Tumor type and grade distribution among lung and pancreas neuroendocrine neoplasia. TC typical carcinoid, AC atypical carcinoid, LCNEC large cell neuroendocrine carcinoma, SCLC small cell lung carcinoma (WHO 2015) [17], NET neuroendocrine tumor, NEC neuroendocrine carcinoma, G grade (WHO 2019) [16]

Figure 1 displays the distribution of the tumor grade stratified by anatomical site. In patients with lung NEN, 61 (72.6%) had a well-differentiated neoplasm with low degree of malignancy, while 23 (27.4%) had a poorly differentiated neoplasms with a high degree of malignancy. Forty-four patients (72.1%) were typical carcinoid (TC) and 17 (20.2%) were atypical carcinoid (AC); seven patients (8.3%) were large cell neuroendocrine carcinomas (LCNEC) and 16 (19.0%) were small cell neuroendocrine carcinomas (SCLC). In patients with pancreatic NEN, 89 (89.0%) had a well-differentiated neoplasm while 11 (11.0%) had poorly differentiated neoplasms with a high degree of malignancy. Fifty-height patients (58.0%) were NET G1, 31 (31.0%) were NET G2 and 11 (11.0%) NEC. No NET G3 was observed.

Patients with both lung and pancreatic NENs were heavier than controls. Pancreatic NEN patients had a higher BMI compared to controls (*p* = 0.033). Patients with lung NEN were more likely to be former or current smokers compared to both controls and to pancreatic NEN. Among patients with pancreatic NEN only, the prevalence of diabetes 2 was significantly higher compared to controls (*p* = 0.002). Both patients with lung and pancreatic NENs reported a significantly higher prevalence of family history of tumor compared to controls (*p* < 0.001 and *p* = 0.001, respectively). Both patients with lung and pancreatic NENs reported a significantly higher prevalence of family history of lung cancer compared to controls.

In Table 2 the Odds Ratios (ORs) and corresponding 95% Confidence Intervals (95% CI) of pancreatic and lung NENs are reported. Increased age at diagnosis was significantly associated with increased risk of lung and pancreatic NENs (OR = 1.03, 95% CI: 1.01–1.05 for both lung and pancreatic NENs). Type 2 diabetes mellitus was associated with an increased risk of pancreatic NEN (OR = 3.01; 95% CI: 1.15–7.89), while history of another tumor other than NENs was associated with an increased risk of both lung and pancreatic NENs (OR = 7.18; 95% CI: 2.55–20.20 and OR = 5.88; 95% CI: 2.43–14.22, respectively). Family history of any tumor and of lung cancer was associated with increased risk of both lung and pancreatic NENs (OR = 2.66; 95% CI: 1.53–4.64 and OR = 2.56; 95% CI: 1.05–6.24 for lung NEN, respectively; OR = 1.94; 95% CI: 1.19–3.17 and OR = 2.60; 95% CI: 1.13–5.95 for pancreatic NEN, respectively). Similar findings were obtained by a separate analysis including only the incident cases (Supplementary Table 1) and in two sensitivity analysis limited to the age-matched participants and to TC and AC cases.

**Table 2:** Odds Ratios and 95% Confidence Intervals for selected covariates in lung and pancreatic neuroendocrine neoplasia

	Lung NEN		Pancreatic NEN		All NENs	
	OR^b^	95% CI	OR^a^	95% CI	OR^b^	95% CI
Height	1.04	0.99–1.08	1.01	0.98–1.05	1.02	0.99–1.06
Weight	1.01	0.99–1.03	1.02	1.00–1.03	1.01	1.00–1.03
BMI						
Underweight	1.00		1.00		1.00	
Normal weight	1.29	0.30–5.60	0.84	0.21–3.39	1.05	0.35–3.17
Overweight	0.95	0.23–3.99	1.06	0.27–4.01	1.01	0.34–2.96
Obese	1.54	0.33–7.13	1.60	0.38–6.74	1.51	0.47–4.80
Smoking status						
Never	1.00		1.00		1.00	
Former	0.79	0.21–2.92	0.59	0.33–1.05	0.91	0.34–2.45
Current	0.82	0.19–3.66	0.59	0.28–1.24	0.91	0.28–2.92
Smoking intensity						
Never	1.00		1.00		1.00	
≤15 pack-years	1.14	0.55–2.35	0.80	0.44–1.46	0.87	0.52–1.47
>15 pack-years	1.65	0.86–3.17	0.43	0.22–1.04	0.84	0.50–1.40
Alcohol status						
Never	1.00		1.00		1.00	
Former	0.81	0.22–2.96	1.31	0.46–3.78	1.36	0.53–3.51
Current	0.97	0.54–1.74	0.78	0.46–1.33	0.90	0.58–1.41
Alcohol consumption						
Never	1.00		1.00		1.00	
≤1 drink per day	0.98	0.52–1.85	0.82	0.46–1.46	0.96	0.59–1.54
>1 drink per day	0.91	0.43–1.95	0.85	0.42–1.71	0.92	0.51–1.67
Diabetes mellitus type 1						
No	1.00		1.00		1.00	
Yes	0.57	0.09–3.45	2.11	0.58–7.70	1.44	0.43–4.84
Diabetes mellitus type 2						
No	1.00		1.00		1.00	
Yes	1.94	0.64–5.87	**3.01**	**1.15**–**7.89**	**2.71**	**1.11**–**6.61**
Other tumor						
No	1.00		1.00		1.00	
Yes	**7.18**	**2.55**–**20.20**	**5.88**	**2.43**–**14.22**	**7.27**	**3.04**–**17.39**
Family history of tumor						
No	1.00		1.00		1.00	
Yes	**2.66**	**1.53**–**4.64**	**1.94**	**1.19**–**3.17**	**2.24**	**1.47**–**3.40**
Family history of lung cancer						
No	1.00		1.00		1.00	
Yes	**2.56**	**1.05**–**6.24**	**2.60**	**1.13**–**5.95**	**2.54**	**1.22–5.30**

Text in bold indicates statistically significant risk factors

*–* not computable

^a^Estimates from multivariate logistic regression adjusted for age, sex and family history of cancer

^b^Estimates from multivariate logistic regression adjusted for age, sex, family history of cancer and smoking intensity

## Discussion

This work contributed novel population-based, case–control data on risk factors associating with neuroendocrine cancer. So far only 11 studies provided similar data (for review see [14, 24, 25]).

Several risk factors were associated with NEN. Among these, the most relevant is the presence of a concurrent tumor (“other tumor”) conferring a significant OR of 7.27 (95% CI 3.04–17.39). This finding well aligns with the two other cancer-related risk factors (“family history of cancer” and “family history of lung cancer”) here identified as significant though with lower ORs (2.24, 95% CI 1.47–3.40 and 2,54 95% CI 1.2–5.3, respectively). Thus, this study provides further evidence that “family history of cancer” is a well-known risk factor for both lung and pancreas neuroendocrine neoplasia. In addition, our data suggest a significant link between neuroendocrine neoplasia and cancer in general, pointing to a cancer predisposition landscape. Genes involved in familial susceptibility are well known for both lung and pancreas NEN. Heritable syndromes associating with NEN development include the multiple endocrine neoplasia type 1 syndrome and, less frequently, the von Hippel-Lindau, the neurofibromatosis type 1, the Cowden, the tuberous sclerosis and the Li-Fraumeni syndromes [26, 27]. During the last three years also deep sequencing data on solid cancer unveiled unexpected germline gene aberrations in neuroendocrine neoplasia (e.g., *MUTYH*, *CHEK2*, and *BRCA2* genes in pancreas NEN) [28, 29] together with a substantial absence of known cancer drivers [30, 31].

Gene-wide association studies (GWAS) are however lacking for patients with neuroendocrine cancer so that more subtle genetic associations have not been elucidated. Though MEN1 patients were not enrolled in this study, we may not exclude the presence of other hidden genetic trait(s) in our cohort. Our results, however, may well represent an epiphenomenon of ageing.

Our data confirm the two major risk factors (“type 2 diabetes” and “family history of cancer”) previously reported for pancreas NEN [14, 24, 25]. Of note, since our data referred to the time prior to the NEN diagnosis, the potential influence of the tumor functionality or its therapy on type 2 diabetes development is excluded. In addition, we found the risk factors “other tumor” and “family history of lung cancer”. Data on secondary tumor and digestive NEN are scant, mainly collected form small series and sometimes incomplete [32–35]. Our finding suggests that in the pancreas some yet undefined cancer predisposition is important for NEN development. On the same line, for lung NEN we confirmed the “family history of cancer” as main risk factor [14], adding “other tumor” as further risk factor. The association of lung neuroendocrine neoplasia and other cancer has been described, but data are limited and usually related to the well-differentiated typical and atypical carcinoid [36]. The concurrent cancer types are those most frequently occurring in the general population, including lung cancer (ibidem). This may well suggest the existence of shared risk factors, including smoking [14]. Previous malignancy has also significant negative impact in lung carcinoid patient survival [37].

The present series reflects the cancer-type distribution of pancreas neuroendocrine neoplasia in the general population (i.e., prevalent well-differentiated tumors and low incident poorly differentiated carcinomas) [38]. Similarly, our series of lung NEN was enriched of the well-differentiated carcinoid groups (about 90% of the whole cases in study), with a relatively low number of poorly differentiated small cell lung carcinoma, a case distribution opposite to that occurring in the general population [17]. This likely reflects both the attraction of NEN patients by the service offered by our referral center and the low number of small cell lung carcinoma survivors recruited for interview along the study period.

This study has some limitations. First, the sample size of the present series may have reduced the actual ability to detect risk factors with low frequency. Of note, the 11 case–control studies published had a similar sample size with only three studies well over 200 cases [24, 39, 40]. Second, the absence of biological details (namely genetics) of investigated cancers precluded further analysis. Lastly, the multicenter source of the present cohort may have introduced center-specific bias.

In conclusion, the present case–control study consistently identified cancer-related variables (“other cancer”, “family history of cancer” and “family history of lung cancer”) as major risk factors for neuroendocrine neoplasia development in pancreas and lung. Our data support the need for further extensive studies on risk factors for neuroendocrine neoplasia.

## Supplementary information

Supplementary information

Supplementary Table 1

## References

[1] Yao JC, Hassan M, Phan A, Dagohoy C, Leary C, Mares JE, Abdalla EK, Fleming JB, Vauthey JN, Rashid A, Evans DB (2008). One hundred years after “carcinoid”: epidemiology of and prognostic factors for neuroendocrine tumors in 35,825 cases in the United States. J. Clin. Oncol..

[2] Dasari A, Shen C, Halperin D, Zhao B, Zhou S, Xu Y, Shih T, Yao JC (2017). Trends in the incidence, prevalence, and survival outcomes in patients with neuroendocrine tumors in the United States. JAMA Oncol..

[3] Korse CM, Taal BG, van Velthuysen ML, Visser O (2013). Incidence and survival of neuroendocrine tumours in the Netherlands according to histological grade: experience of two decades of cancer registry. Eur. J. Cancer.

[4] Hauso O, Gustafsson BI, Kidd M, Waldum HL, Drozdov I, Chan AK, Modlin IM (2008). Neuroendocrine tumor epidemiology: contrasting Norway and North America. Cancer.

[5] Lepage C, Bouvier AM, Phelip JM, Hatem C, Vernet C, Faivre J (2004). Incidence and management of malignant digestive endocrine tumours in a well defined French population. Gut..

[6] Hallet J, Law CH, Cukier M, Saskin R, Liu N, Singh S (2015). Exploring the rising incidence of neuroendocrine tumors: a population-based analysis of epidemiology, metastatic presentation, and outcomes. Cancer.

[7] Tsai HJ, Wu CC, Tsai CR, Lin SF, Chen LT, Chang JS (2013). The epidemiology of neuroendocrine tumors in Taiwan: a nation-wide cancer registry-based study. PLoS ONE.

[8] Huguet I, Grossman AB, O’Toole D (2017). Changes in the epidemiology of neuroendocrine tumours. Neuroendocrinology.

[9] Leoncini E, Boffetta P, Shafir M, Aleksovska K, Boccia S, Rindi G (2017). Increased incidence trend of low-grade and high-grade neuroendocrine neoplasms. Endocrine.

[10] Levi F, Te VC, Randimbison L, Rindi G, La Vecchia C (2000). Epidemiology of carcinoid neoplasms in Vaud, Switzerland, 1974-97. Br. J. Cancer.

[11] Skuladottir H, Hirsch FR, Hansen HH, Olsen JH (2002). Pulmonary neuroendocrine tumors: incidence and prognosis of histological subtypes. A population-based study in Denmark. Lung Cancer.

[12] Lepage C, Bouvier AM, Manfredi S, Dancourt V, Faivre J (2006). Incidence and management of primary malignant small bowel cancers: a well-defined French population study. Am. J. Gastroenterol..

[13] Scherubl H, Streller B, Stabenow R, Herbst H, Hopfner M, Schwertner C, Steinberg J, Eick J, Ring W, Tiwari K, Zappe SM (2013). Clinically detected gastroenteropancreatic neuroendocrine tumors are on the rise: epidemiological changes in Germany. World J. Gastroenterol..

[14] Leoncini E, Carioli G, La Vecchia C, Boccia S, Rindi G (2016). Risk factors for neuroendocrine neoplasms: a systematic review and meta-analysis. Ann. Oncol..

[15] R.V. Lloyd, R. Osamura, G. Kloppel, J. Rosai, WHO Classification of Tumours of Endocrine Organs, vol. 10, 4th edn. WHO Classification of Tumours (IARC Press, Lyon, 2017)

[16] WHO Classification of Tumours Editorial Board. Digestive System Tumours, vol. 1, 5th edn. WHO Classification of Tumours (IARC Press, Lyon, 2019)

[17] W.D. Travis, E. Brambilla, A.P. Burke, A. Marx, A.G. Nicholson, Pathology and Genetics of Tumours of the Lung, Pleura, Thymus and Heart, vol. 7, 4th edn. World Health Organization Classification of Tumours (IARC Press, Lyon, 2015) 10.1097/JTO.0000000000000663 26291007

[18] Decarli A, Franceschi S, Ferraroni M, Gnagnarella P, Parpinel MT, La Vecchia C, Negri E, Salvini S, Falcini F, Giacosa A (1996). Validation of a food-frequency questionnaire to assess dietary intakes in cancer studies in Italy. Results for specific nutrients. Ann. Epidemiol..

[19] Salvini S (1997). A food composition database for epidemiological studies in Italy. Cancer Lett..

[20] Sofi F, Macchi C, Abbate R, Gensini GF, Casini A (2014). Mediterranean diet and health status: an updated meta-analysis and a proposal for a literature-based adherence score. Public Health Nutr..

[21] Nicolotti N, Chuang SC, Cadoni G, Arzani D, Petrelli L, Bosetti C, Brenner H, Hosono S, La Vecchia C, Talamini R, Matsuo K, Muller H, Muscat J, Paludetti G, Ricciardi G, Boffetta P, Hashibe M, Boccia S (2011). Recreational physical activity and risk of head and neck cancer: a pooled analysis within the international head and neck cancer epidemiology (INHANCE) Consortium. Eur. J. Epidemiol..

[22] World Health Organization, Report of a WHO Consultation on Obesity. Obesity: Preventing and Managing the Global Epidemic (World Health Organization, Genenva, Switzerland, 1998) 11234459

[23] K. Rothman, S. Greenland, Modern Epidemiology (Wolters Kluwer Health, Philadelphia, 2008)

[24] Ben Q, Zhong J, Fei J, Chen H, Yv L, Tan J, Yuan Y (2016). Risk factors for sporadic pancreatic neuroendocrine tumors: a case-control study. Sci. Rep..

[25] Valente R, Hayes AJ, Haugvik SP, Hedenstrom P, Siuka D, Korsaeth E, Kammerer D, Robinson SM, Maisonneuve P, Delle Fave G, Lindkvist B, Capurso G (2017). Risk and protective factors for the occurrence of sporadic pancreatic endocrine neoplasms. Endocr. Relat. Cancer.

[26] Aversa JG, De Abreu FB, Yano S, Xi L, Hadley DW, Manoli I, Raffeld M, Sadowski SM, Nilubol N (2020). The first pancreatic neuroendocrine tumor in Li-Fraumeni syndrome: a case report. BMC Cancer.

[27] I. Frayling, M. ARrends, I. Tomlinson, Other genetic tumour syndromes. In: WHO Classification of Tumours Editorial Board (ed.) Digestive System Tumours, vol. 1. WHO Classification of Tumours, p. 550 (IARC, Lyon, 2019)

[28] Dumanski JP, Rasi C, Bjorklund P, Davies H, Ali AS, Gronberg M, Welin S, Sorbye H, Gronbaek H, Cunningham JL, Forsberg LA, Lind L, Ingelsson E, Stalberg P, Hellman P, Janson ET (2017). A MUTYH germline mutation is associated with small intestinal neuroendocrine tumors. Endocr. Relat. Cancer.

[29] Scarpa A, Chang DK, Nones K, Corbo V, Patch AM, Bailey P, Lawlor RT, Johns AL, Miller DK, Mafficini A, Rusev B, Scardoni M, Antonello D, Barbi S, Sikora KO, Cingarlini S, Vicentini C, McKay S, Quinn MC, Bruxner TJ, Christ AN, Harliwong I, Idrisoglu S, McLean S, Nourse C, Nourbakhsh E, Wilson PJ, Anderson MJ, Fink JL, Newell F, Waddell N, Holmes O, Kazakoff SH, Leonard C, Wood S, Xu Q, Nagaraj SH, Amato E, Dalai I, Bersani S, Cataldo I, Dei Tos AP, Capelli P, Davi MV, Landoni L, Malpaga A, Miotto M, Whitehall VL, Leggett BA, Harris JL, Harris J, Jones MD, Humphris J, Chantrill LA, Chin V, Nagrial AM, Pajic M, Scarlett CJ, Pinho A, Rooman I, Toon C, Wu J, Pinese M, Cowley M, Barbour A, Mawson A, Humphrey ES, Colvin EK, Chou A, Lovell JA, Jamieson NB, Duthie F, Gingras MC, Fisher WE, Dagg RA, Lau LM, Lee M, Pickett HA, Reddel RR, Samra JS, Kench JG, Merrett ND, Epari K, Nguyen NQ, Zeps N, Falconi M, Simbolo M, Butturini G, Van Buren G, Partelli S, Fassan M, Khanna KK, Gill AJ, Wheeler DA, Gibbs RA, Musgrove EA, Bassi C, Tortora G, Pederzoli P, Pearson JV, Waddell N, Biankin AV, Grimmond SM, Australian Pancreatic Cancer Genome Initiative (2017). Whole-genome landscape of pancreatic neuroendocrine tumours. Nature.

[30] Priestley P, Baber J, Lolkema MP, Steeghs N, de Bruijn E, Shale C, Duyvesteyn K, Haidari S, van Hoeck A, Onstenk W, Roepman P, Voda M, Bloemendal HJ, Tjan-Heijnen VCG, van Herpen CML, Labots M, Witteveen PO, Smit EF, Sleijfer S, Voest EE, Cuppen E (2019). Pan-cancer whole-genome analyses of metastatic solid tumours. Nature.

[31] The ICGC/TCGA Pan-Cancer Analysis of Whole Genomes Consortium (2020). Pan-cancer analysis of whole genomes. Nature.

[32] Habal N, Sims C, Bilchik AJ (2000). Gastrointestinal carcinoid tumors and second primary malignancies. J. Surg. Oncol..

[33] Verrico M, Rossi L, Tomao S, Colonna M, Vici P, Tomao F (2020). Metachronous and synchronous cancers in patients with neuroendocrine tumors. Oncology.

[34] Kamp K, Damhuis RA, Feelders RA, de Herder WW (2012). Occurrence of second primary malignancies in patients with neuroendocrine tumors of the digestive tract and pancreas. Endocr. Relat. Cancer.

[35] Agasarova A, Harnett C, Mulligan N, Majeed MS, Caimo A, Tamagno G (2018). Management and follow-up of patients with a bronchial neuroendocrine tumor in the last twenty years in ireland: expected inconsistencies and unexpected discoveries. Int. J. Endocrinol..

[36] Cote ML, Wenzlaff AS, Philip PA, Schwartz AG (2006). Secondary cancers after a lung carcinoid primary: a population-based analysis. Lung Cancer.

[37] Filosso PL, Guerrera F, Evangelista A, Welter S, Thomas P, Casado PM, Rendina EA, Venuta F, Ampollini L, Brunelli A, Stella F, Nosotti M, Raveglia F, Larocca V, Rena O, Margaritora S, Ardissone F, Travis WD, Sarkaria I, Sagan D, Committee EN-WS (2015). Prognostic model of survival for typical bronchial carcinoid tumours: analysis of 1109 patients on behalf of the European Association of Thoracic Surgeons (ESTS) Neuroendocrine Tumours Working Group. Eur. J. Cardiothorac. Surg..

[38] Rindi G, Klersy C, Albarello L, Baudin E, Bianchi A, Buchler MW, Caplin M, Couvelard A, Cros J, de Herder WW, Delle Fave G, Doglioni C, Federspiel B, Fischer L, Fusai G, Gavazzi F, Hansen CP, Inzani F, Jann H, Komminoth P, Knigge UP, Landoni L, La Rosa S, Lawlor RT, Luong TV, Marinoni I, Panzuto F, Pape UF, Partelli S, Perren A, Rinzivillo M, Rubini C, Ruszniewski P, Scarpa A, Schmitt A, Schinzari G, Scoazec JY, Sessa F, Solcia E, Spaggiari P, Toumpanakis C, Vanoli A, Wiedenmann B, Zamboni G, Zandee WT, Zerbi A, Falconi M (2018). Competitive testing of the WHO 2010 versus the WHO 2017 grading of pancreatic neuroendocrine neoplasms: data from a large international cohort study. Neuroendocrinology.

[39] Ter-Minassian M, Wang Z, Asomaning K, Wu MC, Liu CY, Paulus JK, Liu G, Bradbury PA, Zhai R, Su L, Frauenhoffer CS, Hooshmand SM, De Vivo I, Lin X, Christiani DC, Kulke MH (2011). Genetic associations with sporadic neuroendocrine tumor risk. Carcinogenesis.

[40] Halfdanarson TR, Bamlet WR, McWilliams RR, Hobday TJ, Burch PA, Rabe KG, Petersen GM (2014). Risk factors for pancreatic neuroendocrine tumors: a clinic-based case-control study. Pancreas.

