# Supplementary material for: Risk factors for pancreas and lung neuroendocrine neoplasms: a case–control study
Source: Endocrine. 2020 Aug 31;71(1):233–41. doi: 10.1007/s12020-020-02464-5 (PMC7835148; doi:10.1007/s12020-020-02464-5)
Supplement: Supplementary file 1 — Supplementary information [file 12020_2020_2464_MOESM1_ESM.pdf]

CODICE **Data Intervista** ………./………./……….

Intervista telefonica …………

Data Diagnosi ………./………./……….

DATI GENERALI

Luogo di nascita:		Data di nascita		
Luogo di residenza		Età		
Sesso<br>(barrare la casella di interesse)	Maschio	Femmina***	***se F compilare sez. F alla fine del questionario	
Stato civile<br>(barrare la casella di interesse)	Celibe/nubile	Sposato/a o convivente	Separato/a o divorziato/a	Vedovo/a
Altezza in cm	Peso in Kg<br>*(Per i CASI indicare il peso prima della diagnosi)*			

PROFESSIONE

Indicare in ordine cronologico le professioni svolte nel corso della propria vita (specificare bene il settore lavorativo)

Professione	Codifica	Da che età	A che età

TITOLO DI STUDIO

Barrare la casella di interesse

Scuola dell’infanzia/Analfabeta	
Scuola elementare	
Scuola media inferiore	
Scuola media superiore	
Laurea di I livello (triennale)	
Laurea di II livello (quinquennale o vecchio ordinamento)	
Scuola di Specializzazione, dottorato	
Altro (specificare)	

FUMO

Nel caso in cui il soggetto abbia smesso e poi ripreso a fumare scrivere per quanti anni ha interrotto/l' anno in cui ha interrotto e l'anno in cui ha ripreso.

Per i CASI specificare 'al momento della diagnosi' (dunque ex fumatore rispetto alla diagnosi).

Attualmente fuma sigarette?	NO	SI		
Ha mai fumato sigarette? (ex fumatore)	NO	SI		
Per quanto tempo?	anni		Età inizio	Età fine
Quante sigarette/die?				

Attualmente fuma sigari?	NO	SI		
Ha mai fumato sigari? (ex fumatore)	NO	SI		
Per quanto tempo?	anni		Età inizio	Età fine
Quanti sigari alla settimana?				

Attualmente fuma la pipa?	NO	SI		
Ha mai fumato la pipa? (ex fumatore)	NO	SI		
Per quanto tempo?	anni		Età inizio	Età fine
Quante volte alla settimana?				

Condivide o ha mai condiviso l'ambiente domestico o lavorativo con fumatori?	NO	SI, per quanto tempo?

CONSUMO DI ALCOL

Specificare anche se l'assunzione è occasionale e indicare quante volte al mese.

Per i CASI specificare 'al momento della diagnosi' (dunque ex bevitore rispetto alla diagnosi).

Attualmente beve **VINO**?	NO	SI	
Ha mai bevuto vino? (ex bevitore)	NO	SI	
Quanti bicchieri a settimana?			
Da e per quanto tempo?	anni	Età inizio	Età fine
In prevalenza vino bianco o rosso?			

Attualmente beve **BIRRA**?	NO		SI
Ha mai bevuto birra?<br>(ex bevitore)	NO		SI
Quanti bicchieri a settimana?			
Da e per quanto tempo?	anni	Età inizio	Età fine

Attualmente beve **BIBITE ALCOLICHE "LEGGERE"** ?<br>(es. Bacardi, smirnoff ...)	NO		SI
Ha mai bevuto BIBITE ALCOLICHE "LEGGERE"?<br>(ex bevitore)	NO		SI
Quanti bicchieri a settimana?			
Da e per quanto tempo?	anni	Età inizio	Età fine

Attualmente beve **SUPERALCOLICI**?	NO		SI
Ha mai bevuto SUPERALCOLICI?<br>(ex bevitore)	NO		SI
Che tipo di superalcolici?			
Quanti bicchieri a settimana?			
Da e per quanto tempo?	anni	Età inizio	Età fine

ATTIVITA' FISICA

Per i CASI le informazioni si devono riferire all'anno precedente la diagnosi

Svolge queste attività fisiche:	N° volte a settimana
Passeggiare all'aria aperta (almeno 20')	
Andare in bicicletta	
Attività sportiva (a livello non agonistico)	
Attività intensa (sport a livello agonistico)	
Curare il giardino/l'orto	
Attività domestiche (lavare, spazzare, stirare, lavori di falegnameria ecc) salire/scendere le scale (ascensore no)	

ABITUDINI ALIMENTARI
Per i CASI le informazioni si devono riferire all'anno precedente la diagnosi

CONSUMA(VA) ABITUALMENTE? (assunzione occasionale = 98)

Pasta o riso (n° porzioni/settimana)	
Pane (n° panini/settimana)	
Polenta (n° fette/settimana)	
Pasticceria (paste, brioches, etc; n°/settimana)	
Carne bovina (n° porzioni/settimana)	
Pollame e coniglio (n° porzioni/settimana)	
Pesce (n° porzioni/settimana)	
Fegato (n° porzioni/settimana)	
Prosciutto crudo (n° porzioni/settimana)	
Prosciutto cotto (n° porzioni/settimana)	
Insaccati (n° porzioni/settimana)	
Carne in scatola (n° porzioni/settimana)	
Latte (n° bicchieri/settimana)	
Formaggio (n° porzioni/settimana)	
Patate (n° porzioni/settimana)	
Legumi (n° porzioni/settimana)	
Totale frutta (n° porzioni/settimana)	
Totale verdura (n° porzioni/settimana)	
Uova (n°/settimana)	

CONSUMA(VA) DURANTE LA STAGIONE APPROPRIATA? (assunzione occasionale = 98)

Cavoli (cavolfiore, verza, broccoli, br.Bruxelles, etc; n° porzioni/settimana)	
Carote (n° porzioni/settimana)	
Spinaci (n° porzioni/settimana)	
Pomodori (n° porzioni/settimana)	
Peperoni (n° porzioni/settimana)	
Insalata verde (n° porzioni/settimana)	
Mele (n° porzioni/settimana)	
Agrumi, spremute incluse (n° porzioni/settimana)	
Melone (n° porzioni/settimana)	

**Consuma(va) PANE INTEGRALE O ALTRI ALIMENTI INTEGRALI?**	Mai	Saltuariamente 1-3 gg/settimana	Abitualmente ≥4 gg/settimana

Come descrive il suo consumo di:	**Scarso**	**Normale**	**Elevato**
Burro			
Margarina			
Olio di oliva			
Olio di semi			

Gradisce e consuma abitualmente cibi:	**Salati**	**Normali**	**Insipidi**
Gradisce e consuma abitualmente cibi e bevande:	**Bollenti**	**Caldi**	**Tiepidi**

Aggiunge alle bevande zucchero (n° cucchiaini/tazza)	
Quanti pasti consuma al giorno? (escludere tè e caffè)	

HA MAI SOFFERTO DI:

Ipertensione arteriosa	NO	SI	Età di insorgenza	
Cardiopatia ischemica (infarto)	NO	SI	Età di insorgenza	
Altre patologie cardiovascolari	NO	SI	Specificare il tipo	Età di insorgenza
Patologie epatiche	NO	SI	Specificare il tipo	Età di insorgenza
Diabete mellito di tipo 1	NO	SI	Età di insorgenza	
Diabete mellito di tipo 2	NO	SI	Età di insorgenza	
Calcoli della colecisti	NO	SI	Intervento chirurgico	NO / SI
Patologie renali	NO	SI	Specificare il tipo	Età di insorgenza
Patologie ematologiche	NO	SI	Specificare il tipo	Età di insorgenza
Patologie polmonari	NO	SI	Specificare il tipo	Età di insorgenza
Fibrosi idiopatica	NO	SI	Età di insorgenza	
Ipertensione polmonare	NO	SI	Età di insorgenza	
Patologie autoimmunitarie	NO	SI	Specificare il tipo	Età di insorgenza
Patologie neurologiche	NO	SI	Specificare il tipo	Età di insorgenza
Patologie gastrointestinali	NO	SI	Specificare il tipo	Età di insorgenza
Tumori	NO	SI	Specificare il tipo	Età di insorgenza
Patologie infettive	NO	SI	Specificare il tipo	Età di insorgenza
Altro (specificare)	NO	SI	Specificare il tipo	Età di insorgenza

E’ mai stato sottoposto a trattamento chemioterapico? Per quale patologia? (specificare)	NO	SI	Durata (specificare il periodo)<br><br>Da (inserire data)<br><br>A (inserire data)	Frequenza (n° volte a sett/mese/anno)
E’ mai stato sottoposto a trattamento radioterapico? Per quale patologia? (specificare)	NO	SI	Durata (specificare il periodo)<br><br>Da (inserire data)<br><br>A (inserire data)	Frequenza (n° volte a sett/mese/anno)

DATI ANAMNESTICI – ASSUNZIONE DI FARMACI

ASSUNZIONE DI ASPIRINA (ACIDO ACETILSALICILICO)

Farmaci che lo contengono:
Acesal, Algo-Nevriton, Alkaseltzer, Ascriptin, Aspiglicina, Aspirina, Aspirinetta, Aspro, Bufferin, Carin, Cebiopirina, Cafiaspirina, Cemerit, Colfarit, Dreimal, Endyol, Fridol, Kilios, Midol, Neocoricidin, Viamal, Vivin

Ha mai fatto uso di aspirina, o di altri prodotti a base di acido acetilsalicilico ALMENO UNA VOLTA ALLA SETTIMANA ? (Registrare solo periodi di utilizzo SUPERIORI A 6 MESI)	SI	NO

PER I “CASI”: PRIMA DELLA DIAGNOSI DI MALATTIA

Frequenza media a settimana	Da che età?	Durata in mesi	Indicazioni<br>1= antidolorifici<br>2= prevenzione cardiovascolare

PER I “CASI”: DOPO LA DIAGNOSI DI MALATTIA

Frequenza media a settimana	Da che età?	Durata in mesi	Indicazioni<br>1= antidolorifico<br>2= prevenzione cardiovascolare<br>3= scopo terapeutico post diagnosi

PER I “CONTROLLI”

Frequenza media a settimana	Da che età?	Durata in mesi	Indicazioni<br>1= antidolorifici<br>2= prevenzione cardiovascolare

Ha mai subito nella sua vita **RICOVERI IN OSPEDALE?**	NO	SI (specificare motivo)
		SI (specificare motivo)
		SI (specificare motivo)

Ha assunto farmaci per ALMENO 6 MESI? Se sì, quali?

PER L'INTERVISTATORE, citare:.
Antiacidi, Inibitori della pompa protonica, Antibiotici, Diuretici, Beta-bloccanti, Calcioantagonisti, FANS (esclusa aspirina), Tranquillanti, Statine, Metformina, Altri ipoglicemizzanti orali, Insulina

Tipo di farmaco	Nome farmaco	Durata (specificare se MESI o ANNI)	ETA’ al primo utilizzo

ASSUNZIONE DI STUPEFACENTI

Ha mai assunto le seguenti sostanze?			Frequenza di uso	Durata (specificare se MESI o ANNI)	ETA' al primo utilizzo
Cannabis: hashish e marijuana	NO	SI			
Cocaina o Crack	NO	SI			
Sostanze stimolanti sintetiche (ecstasy, amfetamina, metamfetamina, "pasticche")	NO	SI			
Eroina, metadone, morfina	NO	SI			
Altra sostanza (specificare)					
Altra sostanza (specificare)					
Altra sostanza (specificare)					

ANAMNESI FAMILIARE

Quante sorelle ha avuto?	
Quanti fratelli ha avuto?	
Quante figlie ha avuto?	
Quanti figli ha avuto?	
Quanti coniugi/conviventi ha avuto?	

Malattie genetiche note:

MEN I	SI	NO
MEN II	SI	NO
Altra (specificare)		
Altra (specificare)		

Completetare le colonne dello schema seguente per ciascun membro della famiglia, indicando per ciascuno eventuali tumori:

madre [1] sorella [3] figlia [5] coniuge [7]
padre [2] fratello [4] figlio[6]

PARENTE	Vivo=1 Morto=2	ETA' attuale o alla morte	TUMORE Si=1, No=2, Non so=3 (specificare la sede)		Codice ICD IX	ETA' ALLA DIAGNOSI
[1]						
[2]						

SEZIONE F: SOLO PER LE PAZIENTI DI SESSO FEMMINILE

Età al menarca	
**CICLI MESTRUALI**	
Durata in giorni della perdita ematica	
Durata media cicli mestruali (escluso durante l’assunzione di contraccettivi orali)<br><br>(Barrare la casella di interesse)	< 21 giorni
	21-25 giorni
	26-30 giorni
	31-35 giorni
	> 35 giorni
	Totalmente irregolari
	Non so

Stato menopausale<br><br>(Barrare la casella di interesse)	pre
	peri
	post-menopausa

Se in post-menopausa:	età alla menopausa:
Tipo di menopausa:<br><br>(Barrare la casella di interesse)	naturale
	chirurgica
	da raggi
	farmacologica

Numero totale di:	
Nati	
Aborti spontanei	
Interruzioni volontarie	
Età alla prima gravidanza (aborto o parto)	

La prima gravidanza è terminata in:<br><br>(Barrare la casella di interesse)	aborto spontaneo
	aborto volontario
	nato morto
	nato vivo

Età a ciascun parto

(Gravidanza portata a termine, sia con un nato vivo, sia con un nato morto)

I parto	II parto	III parto	IV parto	V parto	VI parto

Allattamento

(Indicare il numero di mesi/barrare la casella di interesse)

**Allattamento** (Barrare la casella di interesse)	I parto	II parto	III parto	IV parto	V parto	VI parto
non ha allattato per ragioni personali o mediche						
mesi di allattamento (indicare il numero, da 1 a 6, se >6 indicare 6						
ha allattato ma non ricorda per quanti mesi						
ha tentato ma non è riuscita ad allattare						
Non ricorda						

Alla prima gravidanza, quanti mesi ha trascorso senza usare alcuna precauzione, prima di rimanere incinta?	Indicare il numero di mesi:

E' stata mai in cura per la sterilità? NO SI a quale età ?	
Per quale causa? (Barrare la casella di interesse)	Occlusione tubarica
	Squilibri ormonali
	Endometriosi
	Sterilità del partner
	Altro (specificare)

DATI ANAMNESTICI – ASSUNZIONE DI FARMACI

Ha assunto i seguenti farmaci per ALMENO 6 MESI? (Se si specificare il nome commerciale)			Nome farmaco	Durata (specificare se MESI o ANNI)	ETA' al primo utilizzo
Terapia ormonale sostitutiva	NO	SI			
Tamoxifene	NO	SI			
