# Supplementary material for: Risk factors for pancreas and lung neuroendocrine neoplasms: a case–control study
Source: Endocrine. 2020 Aug 31;71(1):233–41. doi: 10.1007/s12020-020-02464-5 (PMC7835148; doi:10.1007/s12020-020-02464-5)
Supplement: Supplementary file 2 — Supplementary Table 1 [file 12020_2020_2464_MOESM2_ESM.pdf]

Supplementary Table 1 - Odds Ratios and 95% Confidence Intervals for lung and pancreatic neuroendocrine neoplasia among incident cases

	Lung NEN		Pancreatic NEN		All NENs	
	OR[b]	95% CI	OR[a]	95% CI	OR[a]	95% CI
Height	1.03	0.98-1.07	1.02	0.98-1.06	1.03	0.99-1.06
Weight	1.01	0.99-1.03	1.02	1.00-1.04	1.01	1.00-1.03
BMI						
underweight	1.00		1.00		1.00	
normal weight	1.08	0.24-4.75	0.73	0.18-3.02	0.88	0.29-2.70
overweight	0.81	0.19-3.43	0.87	0.22-3.41	0.83	0.28-2.46
obese	1.41	0.30-6.66	1.55	0.37-6.60	1.42	0.44-4.56
Smoking status						
never	1.00		1.00		1.00	
former	0.51	0.12-2.20	0.54	0.29-1.00	0.75	0.26-2.14
current	0.51	0.10-2.63	0.61	0.28-1.32	0.78	0.23-2.65
Smoking intensity						
never	1.00		1.00		1.00	
≤15 pack-years	0.98	0.44-2.19	0.78	0.41-1.48	0.80	0.46-1.39
>15 pack-years	1.89	0.95-3.76	0.39	0.19-1.08	0.86	0.50-1.47
Alcohol status						
never	1.00		1.00		1.00	
former	0.69	0.17-2.74	1.35	0.46-3.92	1.33	0.50-3.50
current	1.03	0.56-1.91	0.58	0.32-1.03	0.79	0.50-1.26
**Alcohol consumption**						
never	1.00		1.00		1.00	
≤ 1 drink per day	1.12	0.58-2.18	0.63	0.34-1.17	0.88	0.54-1.46
>1 drink per day	0.78	0.34-1.80	0.68	0.32-1.43	0.75	0.40-1.41
Diabetes mellitus type 1						
no	1.00		1.00		1.00	
yes	0.68	0.11-4.14	2.53	0.69-9.29	1.73	0.51-5.85
Diabetes mellitus type 2						
no	1.00		1.00		1.00	
yes	2.03	0.64-6.46	**3.60**	**1.37-9.48**	**3.10**	**1.26-7.64**
Other tumor						
no	1.00		1.00		1.00	
yes	**7.13**	**2.40-21.20**	**5.16**	**2.06-12.96**	**6.81**	**2.77-16.72**
Family history of tumor						
no	1.00		1.00		1.00	

yes	**2.65**	**1.47-4.78**	**2.10**	**1.25-3.54**	**2.36**	**1.52-3.66**
Family history of lung cancer						
no	1.00		1.00		1.00	
yes	2.17	0.81-5.83	**2.66**	**1.12-6.32**	**2.40**	**1.11-5.17**

[a] Estimates from multivariate logistic regression adjusted for age, sex and family history of cancer
[b] Estimates from multivariate logistic regression adjusted for age, sex, family history of cancer and smoking intensity

Text in bold indicates statistically significant risk factors

- not computable
